# Quality of Life in Patients with Functional Dyspepsia: A Comparative Study

**DOI:** 10.34172/mejdd.3399

**Published:** 2026-04-30

**Authors:** Zahra Momayez Sanat, Fatemeh Majidirad, Nasim Ebadati Nakhjiri, Ali Montazeri

**Affiliations:** ^1^Digestive Diseases Research Institute, Shariati Hospital, Tehran University of Medical Sciences, Tehran, Iran; ^2^Department of Physical Therapy, Faculty of Rehabilitation Sciences, Tehran University of Medical Sciences, Tehran, Iran; ^3^Department of Family Medicine, School of Medicine, Ziaeian Hospital, Tehran University of Medical Sciences, Tehran, Iran; ^4^Health Metrics Research Center, Institute for Health Sciences Research, ACECR, Tehran, Iran; ^5^Faculty of Humanity Sciences, University of Science and Culture, Tehran, Iran

**Keywords:** Functional dyspepsia, Quality of life (QoL), Short form 36 (SF36) questionnaire

## Abstract

**Introduction:**

Dyspepsia severely affects patients’ well-being and quality of life. The effect of psychosocial factors on symptom severity and generation, particularly their impact on clinical management, warrants further investigation. Thus, this study aimed to assess quality of life (QoL) in patients with functional dyspepsia.

**Methods:**

This was a comparative study on a sample of patients with dyspepsia and a comparison group. The diagnosis of dyspepsia was based on the Rome IV criteria, and the Short Form Health Survey (SF-36) was used to compare QoL between the study samples by performing analysis of covariance (ANCOVA).

**Results:**

The patient group had a lower QoL, and *P* values were significant in all subscales and components. Pearson’s Chi-square test was significant only for pain-relief medication use (*P*<0.001). *P* value of Pearson’s Chi-square was 0.054 and 0.88, respectively, for smoking and alcohol use variables.

**Conclusion:**

The findings demonstrated that functional dyspepsia affects both physical and psychological health and decreases QoL.

## Introduction

 Dyspepsia symptoms are characterized by upper abdominal complaints, such as early satiety, postprandial fullness, epigastric burning, epigastric pain, bloating, feeling of fullness, and nausea. Functional dyspepsia or non-ulcer dyspepsia (NUD) can be diagnosed when the organic cause of the symptoms cannot be recognized, and patients undergo a formal endoscopic or radiographic evaluation without an organic lesion (ulcer, or tumor) to explain their symptoms.^1,2^ Functional dyspepsia can be defined using Rome IV criteria and subcategorized into epigastric pain syndrome and postprandial distress syndrome, considering the predominance of post-prandial bloating and epigastric versus fullness pain.^1,3^

 The prevalence of dyspepsia in the community ranges from 20% to 40%, and about 50% to 60% are sub-classified as functional dyspepsia.^3^ The prevalence of dyspepsia in Asians has been declared to be 8% to 30%, while the rate of functional dyspepsia has been reported as 8%-23%.^4^ Most investigations have declared that women have higher dyspepsia prevalence. Dyspepsia is very common in Iran.^5^

 Its possible causes include gastroduodenal dysmotility, gastric acid hypersecretion, emotional stress, visceral hypersensitivity, and psychological factors. Also, the possibility of underlying lifestyle and psychosocial factors should be considered. The functional dyspepsia heterogeneity can be approved by the involvement of one or more of such disturbances in some cases.^2,6^

 The most common psychiatric comorbidities in patients with functional dyspepsia are depression, anxiety, somatoform diseases, and a remote or recent history of sexual or physical abuse. Psychological distress is a healthcare-seeking behavior feature in cases with functional bowel diseases, such as functional dyspepsia.

 These results indicate a relationship between various psychosocial factors and the severity and presence of symptoms in functional dyspepsia, but this relationship does not seem to be simple. For example, epigastric pain is associated with visceral hypersensitivity, many psychosocial dimensions (such as neuroticism and somatization), and low health-related quality of life (HRQoL).

 Therefore, a relationship is observed between visceral hypersensitivity and psychosocial factors. A relationship is observed between gastric sensitivity and anxiety in patients (the subgroup of hypersensitive patients) with functional dyspepsia. A relationship is observed between a history of sexual or physical abuse and visceral hypersensitivity in patients with functional dyspepsia.

 Dyspepsia, particularly functional dyspepsia, significantly affects the quality of life (QoL). Factors, such as fear of an underlying severe disease, symptom severity, not enough psychosocial support, and psychological distress, influence health care seeking.^2^ Regardless of the cause, dyspepsia negatively influences patients’ HRQoL and leads to a marked economic burden.

 The effect of psychosocial factors on symptom severity and generation, particularly their impact on clinical management, merits further investigation. Thus, this study was designed and implemented to compare the QoL in patients with functional dyspepsia and a comparison group using the short form 36 (SF-36) questionnaires in Iran. We hypothesized that QoL in patients with functional dyspepsia is impaired compared with that of the general population.

## Materials and Methods

###  Design 

 This study employed a cross-sectional design to compare QoL between a sample of patients with dyspepsia and individuals attending the ear, nose, and throat (ENT) and eye clinics as a comparison group. A trained psychologist completed the SF-36 questionnaires for all participants. The Ethics Committee of Tehran University of Medical Sciences approved the study (IR.TUMS.MEDICINE.REC.1400.257), and written consent was obtained from all participants.

###  Participants

 The research population included all patients with medical records referred to the gastrointestinal clinic of a teaching hospital affiliated with Tehran University of Medical Sciences, in Tehran, Iran, in 2019 and 2020. The case group included outpatients with functional dyspepsia enrolled by a gastroenterologist, and the comparison group comprised patients attending the ENT and eye departments. In general, care was taken to ensure that individuals with chronic and serious medical conditions were not included in the study. Moreover, many of these individuals were healthy subjects or patients’ companions.

###  Inclusion and Exclusion Criteria

 All cases diagnosed based on the Rome IVcriteria and clinical evaluation were included. Patients were included with confirmed epigastric or upper abdominal manifestations (discomfort or pain) lasting at least three continuous months or alternatively during the past year. Patients should be aged 18 years or above. There was no sex restriction. Patients with a history of psychological problems (such as major depression or known psychiatric illnesses) who were under the care of a psychiatrist or psychologist, or taking antidepressant medication, and organic dyspepsia with an identified cause for the symptoms (peptic ulcer disease, erosion, gastropathy, and duodenopathy) were excluded. The comparison group was included if they were over 18 years of age, had no debilitating disease or a history of cancer or malignancy, and had no history of depression or anxiety.

###  Measure

 The short form Health Survey (SF-36) was used to assess HRQoL. It is a well-recognized instrument containing 36 items tapping into eight subscales: physical functioning (PF), role physical (RP), bodily pain (BP), general health (GH), social functioning (SF), vitality (VT), role emotional (RE), and mental health (MH). It also provides two summary scores for mental and physical components (PCS and MCS). The scores for all subscales and two summary components range from 0 (the worst) to 100 (the best) conditions. We used the Iranian version of the questionnaire. The psychometric properties of the Iranian version showed satisfactory results.^7^

###  Sampling and Sample Size

 Considering a study with 80% power at the 5% significance level to detect a 20% difference in QoL scores between the two study groups, a minimum sample size of 100 participants per group was estimated. However, in practice, 172 patients from the eye clinic and 137 patients with functional dyspepsia were entered into the study.

###  Statistical Analysis

 Data were analyzed using SPSS software version 23. According to the missing observations, the maximum range of data was included. The Kolmogorov-Smirnov test was used to assess normality. An independent sample t-test was used for comparison. Since there were differences between the study groups in demographic characteristics, we performed an analysis of covariance (ANCOVA) to adjust for these differences. A *P* value of less than 0.05 was considered significant.

## Results

 A total of 309 patients (172 patients in the comparison group and 137 patients with functional dyspepsia). The distribution of sex in each group, demographic characteristics, and variables, including smoking, alcohol use, and pain relief medication use separately for the comparison group and patient group, were shown in Table 1.

**Table 1:** Characteristics of the study samples

**Variables**	**Comparison group ** **(n=172)**	**Patients ** **(n=137)**	***P*** *
Age (mean, SD)	37.02 (10.3)	45.06 (13.0)	< 0.001
Sex (No., %)			0.153
Male	38 (22.1)	40 (29.2)	
Female	134 (77.9)	97 (70.8)	
BMI (mean, SD)	26.5 (4.17)	27.9 (6.18)	0.019
Smoking (No., %)			0.195
No	155 (90.1)	129 (94.2)	
Yes	17 (9.9)	8.0 (5.8)	
Alcohol (No., %)			0.961
No	163 (94.8)	130 (94.9)	
Yes	9.0 (5.2)	7.0 (5.1)	

* Derived from t-test or Chi-square as applied.

 As shown, age and BMI differed significantly between the two groups (*P*= 0.001 and 0.014, respectively); therefore, ANCOVA was used to compare SF-36 scores between the two study groups. Table 2 presents the comparison of eight subscales and two components of the SF-36 questionnaire. In all instances, the patient group had lower QoL, and *P* values were significant.

**Table 2:** Comparison of quality of life scores between the study samples

**Variables**	**Comparison group (n=172)**	**Patients ** **(n=137)**	
	**Mean (SD)**	**Mean (SD)**	***P*** *
Physical functioning	56.82 (10.92)	46.00 (17.11)	< 0.001
Role physical	81.83 (28.87)	61.31 (41.45)	< 0.001
Bodily pain	75.19 (21.70)	53.12 (23.94)	< 0.001
General health	65.46 (17.16)	50.69 (21.45)	< 0.001
Vitality	64.24 (18.20)	57.15 (17.63)	0.010
Social functioning	69.84 (20.89)	61.95 (17.86)	0.007
Role emotional	72.09 (38.94)	50.85 (45.20)	0.001
Mental health	67.37 (17.00)	61.51 (14.74)	0.006
Mental component summary	48.03 (10.97)	43.75 (11.02)	0.011
Physical component summary	46.72 (6.29)	40.44 (8.73)	< 0.001

*Derived from ANCOVA adjusted for age and BMI as covariates

## Discussion

 This study demonstrates that QoL among individuals with dyspepsia was significantly lower than that of the comparison group across all components of the SF-36 questionnaire.

 Consistent with our findings, Laima Alam and colleagues reported that patients with functional dyspepsia had a low QoL in all areas affecting emotional, physical, and psychological health.^8^ Additionally, Aro and co-workers highlighted the negative impact of this condition on the physical, psychological, and social aspects of QoL.^9^

 In a previous European study, the findings showed that symptom relief in patients with functional dyspepsia positively affected life quality and reduced subsequent costs for three months after the discontinuation of initial treatment.^10^

 In another study, the QoL was decreased in patients with functional dyspepsia, and improvements were observed as symptom severity decreased during a one-year follow-up.^11^ Consistent with our study, Halling et al.’s study reported that dyspepsia and decreased vitality were the main factors for decreasing the QoL.^12^

 Our study showed that patients with functional dyspepsia had lower scores on the mental component of QoL. The results of a meta-analysis indicated a moderate correlation between depressive dyspepsia and anxiety, and the dyspepsia group had significantly more frequent periods of depression and anxiety disorder than the control group.^13^ We suggested that anxiety and depression could decrease the threshold of pain in patients with functional dyspepsia, which could reduce QoL in these patients.

 Filipović and others observed that depression and anxiety levels were high in patients with functional dyspepsia. Psychological disorders can affect the QoL, especially in patients with dyspepsia, in the form of emotional distress, and difficulty in consuming food and beverages affects QoL.^14^

 Another study has indicated that the sensory-motor function of the stomach is not significantly associated with poor QoL, while personality traits and depressed mood strongly affect the QoL.^15^ In contrast, Halling and co-workers emphasized abdominal pain and dyspepsia, problems with food and beverage intake, and decreased vitality as major factors that deteriorate QoL. Furthermore, the effect of this disease on QoL is comparable to the effect of “organic” chronic diseases, such as asthma and inflammatory bowel disease.^14^

 Also, Mahadeva and colleagues showed that patients with functional dyspepsia had a lower QoL than patients with organic diseases, but this could not be attributed to differences in anxiety or depression between the two groups.^16^ A 12-year cohort study revealed that major depressive disorder or anxiety disorder is a risk factor for new-onset irritable bowel syndrome (IBS). Conversely, gastrointestinal dysfunction, including IBS and functional dyspepsia, has been reported as a risk factor for new-onset depressive disorder or anxiety disorder. These findings indicate the pathophysiological connections of the brain to the intestine and the intestine to the brain.^17^

 Hantoro and colleagues reported that anxiety, depression, old age, female sex, higher symptom severity, and low to moderate education level were significant factors associated with low HRQoL.^18^

 Measuring the QoL is essential to understanding the effects of chronic diseases. Determining the disease burden using traditional criteria, such as disease prevalence and direct/indirect costs, is not enough to understand the actual burden of any disease. Identifying the adverse effects of a disease on QoL is vital to the community, and healthcare providers are fully aware of the disease’s actual burden. Disease-related QoL data can also help national health policymakers when budgeting for healthcare.^19^

 The significant decrease in the SF-36 QoL scores in this study, even after excluding participants who have been diagnosed with psychiatric conditions, is consistent with current literature. This serves as evidence that low QoL in patients with functional dyspepsia is not only a result of the presence of coexisting psychiatric illness (such as anxiety or depression, etc.) but is also influenced by the disease’s inherent pathogenesis. Indeed, despite the lack of a clinical diagnosis for any psychiatric disease, the disease is linked to subtle mental disturbance in the form of psychophysiological processes involving visceral hypersensitivity and disturbances in brain-gut axis processing, hence negatively affecting the SF-36 mental component measures. The physical component of the SF3 questionnaire could also be directly affected by the functional dyspepsia.

## Conclusion

 In conclusion, this study showed that functional dyspepsia affects both physical and psychological health and reduces QoL. Therefore, further studies are recommended to explore strategies to improve QoL in these patients.
